# Preparation, Characterization of Pregabalin and *Withania coagulans* Extract-Loaded Topical Gel and Their Comparative Effect on Burn Injury

**DOI:** 10.3390/gels8070402

**Published:** 2022-06-24

**Authors:** Anam Asghar, Muhammad Naeem Aamir, Fatima Akbar Sheikh, Naveed Ahmad, Nasser F. Alotaibi, Syed Nasir Abbas Bukhari

**Affiliations:** 1 Department of Pharmaceutics, Faculty of Pharmaceutical Sciences, Government College University Faisalabad, Faisalabad 38000, Pakistan; dr.anumasghar@yahoo.com; 2 Department of Pharmaceutics, Faculty of Pharmacy, The Islamia University of Bahawalpur, Bahawalpur 63100, Pakistan; 3 School of Pharmacy, Newcastle University, Newcastle upon Tyne NE1 7RU, UK; 4 Al-Raziq College of Pharmacy, Sargodha 40100, Pakistan; fatimatahir303@gmail.com; 5 Department of Pharmaceutics, College of Pharmacy, Jouf University, Sakaka 72388, Saudi Arabia; nakahmad@ju.edu.sa; 6 Chemistry Department, College of Science, Jouf University, Sakaka 72388, Saudi Arabia; nfalotaibi@ju.edu.sa; 7 Department of Pharmaceutical Chemistry, College of Pharmacy, Jouf University, Sakaka 72388, Saudi Arabia; sbukhari@ju.edu.sa

**Keywords:** pregabalin, *Withania coagulans*, co-combination gel, topical delivery, burn injury

## Abstract

The current study depicts the comparative effects of nanogel using *Withania coagulans* extract, pregabalin alone, and a co-combination gel. The gels prepared were then analyzed for conductivity, viscosity, spread ability, globule size, zeta potential, polydispersity index, and TEM. The globule size of the co-combination gel, determined by zeta sizer, was found to be (329 ± 0.573 nm). FTIR analysis confirms the successful development of gel, without any interaction. Drug distribution at the molecular level was confirmed by XRD. DSC revealed no bigger thermal changes. TEM images revealed spherical molecules with sizes of 200 nm for the co-combination gel. In vivo studies were carried out by infliction of third degree burn wounds on rat skin, and they confirmed that pregabalin and *Withania coagulans* heals the wound more effectively, with a wound contraction rate of 89.95%, compared to remaining groups. Anti-inflammatory activity (IL-6 and TNF-α), determined by the ELISA technique, shows that the co-combination gel group reduces the maximum inflammation with TNF-α value (132.2 pg/mL), compared to the control (140.22 pg/mL). Similarly, the IL-6 value was found to be (78 pg/mL) for the co-combination gel and (81 pg/mL) in the case of the control. Histopathologically, the co-combination gel heals wounds more quickly, compared to individual gel. These outcomes depict that a co-combination gel using plant extracts and drugs can be successfully used to treat burn injury.

## 1. Introduction

Wound healing is a complicated process, in which tissue repairs itself after injury [1]. Burn injuries are considered one of the most destructive injuries in the world and are associated with inflammation, neuropathic pain, nerve/tissue damage, infection, disability, and mortality [2].

Burn pain can be considered neuropathic, as it results in simultaneous inflammation and central sensitization [3]. A deep partial thickness is an excellent burn wound healing model, in regard to studying the three main components of wound healing, i.e., epithelialization, contraction, and scar formation. Different investigators used different burn models and treatment approaches [4]. The burn-induced pain was confirmed by mechanical responses after burn injury [5].

The use of herbal products, as safer remedies, has been reported in the literature. The herbal product reduces wound repair time and affects various phases of wound healing, such as coagulation, inflammation, epithelization, collaganation, and wound contraction [6]. Different medicines have been used, along with other medicines and many non-invasive techniques for topical treatment [7]. According to the WHO, 265,000 deaths occur annually due to burning. Microbial nutrient availability, skin barrier disruption, vascular supply destruction, and systemic immune-suppressants are the parameters susceptible to wound infection. Topical antimicrobials, pain suppressants, dressing, systemic antibiotics, and burn therapy are the methods used for treatment [8]. However, systemic medicines face problem in reaching the infected sites, suffer antimicrobial resistance, and have systemic side effects. The use of nanotechnology, along with plant extracts, in order to have a synergistic effect and better outcome, was evaluated in the current study. Co-combination gel incorporating pregabalin and plant extracts simultaneously in a polymeric network has not yet been reported in the literature.

There are two majorly used gabapentinoid drugs for treating hyperalgesias, namely gabapentin and pregabalin [9]. Multimodal analgesia is appropriate for the pain [1]. Pregabalin is an antiepileptic drug and analogue of gabapentin. Pregabalin binds to the alpha-2-delta 1 subunit of presynaptic voltage-gated calcium channels [10]. Pregabalin has been used in the treatment of postoperative pain and burn injury in previous studies, both alone or in combination with other drugs [11].

*Withaniacoagulans* have been used as a cure for a plethora of diseases and conditions. *Withania coagulans*, known as paneer doda, belong to the family Solanaceae, found in Pakistan and its surroundings. Active constituents *Withaferin, Withanolide,* and *Withacoagin* [12] have been isolated from *Withania coagulins* fruits. The twigs are used for teeth cleaning, and the smoke of the plants is inhaled for relieving tooth pain [13]. The fruit part is used for the treatment of asthma and liver disorders. It possesses anti-nociceptive, -microbial, -fungal, and -inflammatory properties [14]. In diabetes induced neuropathy and nephropathy [15], it is also used as a hepatoprotective agent [16]. It has been used in breast cancer for its apoptotic activity [17]. In diabetes, it is also used in lowering glucose levels [18]. In rheumatoid arthritis/arthritis, *Withania coagulans* have been found very useful [19].

## 2. Materials and Methods

### 2.1. Preparation of Pregabalin and Withania coagulans Topical Gel

**Figure 1:**
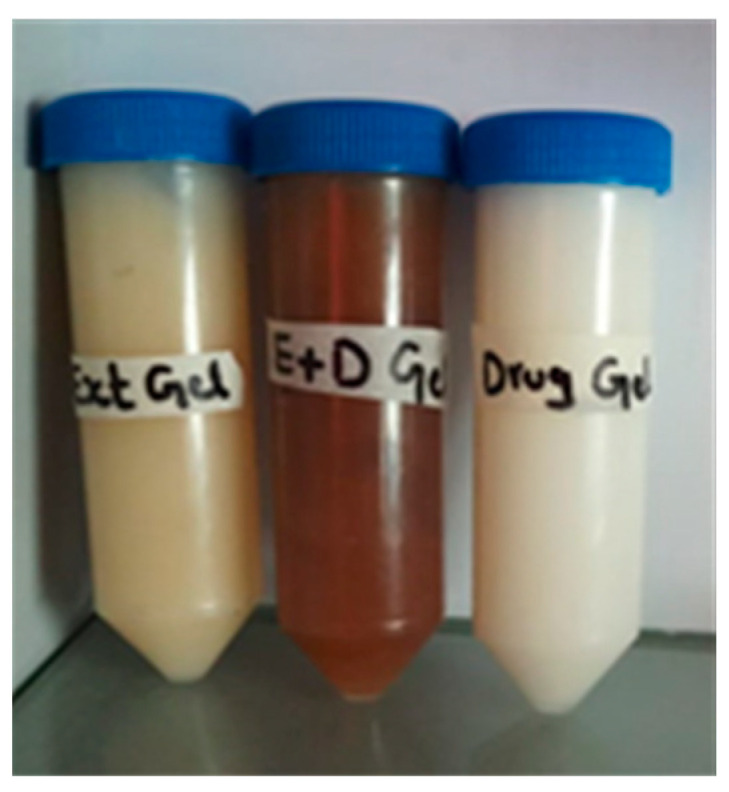
Topical Nanogels.

Complete details of gel preparation are given [20] briefly. Microemulsion were prepared by pseudo ternary phase diagram, using Chimax school software version 8. Pregabalin topical gel 2.5% and *Withania coagulans* gel 2% were prepared using 1% Carbopol 934 [20], and pH was adjusted to 5.5 by triethanolamine [21]. The gels prepared were applied topically twice a day at 8 am and 12 pm on burn wounds. The diameter of the wound was calculated before and after the application of the topical gel on days 0, 3, 7, 9, 11, 17, and 21. Table 1 and Figure 1 show the composition of pregabalin and *Withanai coagulans* topical gel.

#### 2.1.1. Viscosity Measurements

The viscosity of gels was measured by rotational viscometer (Brookfield DV-II +Pro UK) at 25 ± 0.5 °C. The speed was kept at 100 rpm, using 6 spindle sizes for 1 min.

#### 2.1.2. pH

The pH is an important parameter to judge the irritability of topical formulations. The pH of all three gels was measured using a pH meter (HI 2210 Hanna, Woonsocket, RI, USA).

#### 2.1.3. Conductivity

Electrical conductivity is an important parameter for finding out the external/internal phase of nanogels. The conductivity of all nanogels was measured using a conductivity meter (EcoScan, con5, Eutech instrument, Paisley, UK).

#### 2.1.4. Spreadability

Spreadability was checked by placing 1 g of gel between two glass slides. A weight of 250 g was placed over the 2nd slide, and the area of gel spread ability was noted. Spreadability was calculated by the formula [22,23].

S = W_1_ − W_2_/W_1_ × 100 (i)

#### 2.1.5. Particle Size Determination

Particle and globule sizes are an important parameter for finding out whether the size falls within the microgels, macrogels, or nanogels range. Average droplet size, polydispersity index, and zeta potential of all gels was measured using a photon correlation spectrophotometer (Malvern Zetasizer). The gel samples were placed in a cuvette in the thermostatic chamber. The samples were diluted with distilled water, and readings were taken.

#### 2.1.6. Drug/Extract Content Determination

*Drug* and *Withania coagulans* λmax were determined by preparing a stock solution of the extract by dissolving 1 g in 100 mL methanol. A calibration curve was plotted after taking absorbance against different concentrations. For drug content determination, 1 g of all gel samples was taken and diluted with methanol. The mixture was mixed by vortex and then sonicated for 2 min in an ultrasonicator. Then, absorbance was taken λmax at 294.5 nm for the drug and 340 nm for the extract-loaded gel. The measurements were taken in triplicate.

#### 2.1.7. Characterization of Nanogels

The prepared gel was characterized through FTIR, DSC, XRD, and TEM.

#### 2.1.8. Drug–Excipient Compatibility Studies

FTIR technique was used to study physical and chemical interaction between drug and excipients. A 100 mg of sample was mixed thoroughly with potassium bromide and compacted under vacuum pressure of 12 psi for 3 min. The disc was mounted in a suitable holder in a Perkin Elmer IR spectrophotometer, and spectra were recorded from 2000–400 cm^−1^. The resultant spectra of pure drug were compared with spectra of its physical mixture.

#### 2.1.9. DSC and TG Analysis

The thermal and phase transition behavior of pregabalin, *Withania coagulans* extract, and prepared gels were determined by DSC technique with a nitrogen purging. The heating rate of 20 °C/min was employed over a temperature range of 50–250 °C. A slander aluminum sample pan was used.

#### 2.1.10. X-ray Diffraction of Gels

Samples were placed in a special plane glass. Small angle X-ray diffraction was observed by using a super speed diffractometer with a Ni filter, Cu radiation, tube voltage 25–45 KV, and tube current 100–200 mA, scanned from 2° to 70°, 2θ.

#### 2.1.11. Transmission Electron Microscopy

A total of 10 folds aqueous diluted samples were subjected to collodion-coated 300 mesh copper grid and kept for 5 min adsorbed using a filter paper; then, a drop of 2% aqueous uranyl acetate was applied for 1 min. The remaining solution was removed, and samples were air dried and examined at 200 KV.

### 2.2. Burn Wound Healing Studies

#### 2.2.1. Animal

A total of 15 Albino rats, weighing 200–300 kg, were housed in a separate cage for 1 week before study. All rats were provided with food and water *ad libitum.* Rats were weighed daily.

#### 2.2.2. Skin Clipping

A total of 24 h before burn injury, rats were anesthetized with ketamine HCl 50 mg/kg and xylazine 20 mg/kg intraperitoneally, and the back skin was clipped using an electrical clipper and depilatory cream. This procedure was adopted to remove the fur of animals uniformly. Clipping and depilating animal skin can produce rashes on the skin; so, it is recommended that 24 h should be elapsed before the burn is inflicted.

#### 2.2.3. The Burn Injury

The heated template was applied to the back of the animal skin for 30 s using analogue stopwatch. Only minimal pressure was applied between the template and animal skin, in order to ensure perfect contact between the two. The soldering method was used. Cylindrical iron templates (diameter 2.4 cm, height 12.5 cm, length 11 cm of handle, and total weight 270 g) were heated on an electrical heater to generate a heat of 100 °C for 45 s. A total of 5–6 templates were heated in a similar manner, in order to be used alternatively on each animal for 30 s [24].

#### 2.2.4. Infliction of Wound

The heated template was applied to the back of the animal skin for 30 s using analogue stopwatch. Only minimal pressure is applied between the template and animal skin, in order to ensure perfect contact between the two. After 30 s, heat source was removed. The same procedure was repeated for all animals groups.

### 2.3. Animal Grouping

Rats were divided into five groups (n = 6 each)

1. No treatment group (control);
2. Topical application with *Withania coagulans* extract gel;
3. Topical application with pregabalin drug solution;
4. Topical application with pregabalin gel;
5. Topical application of pregabalin and *Withania coagulans* combined gel.

#### Burn Injury Treatment

Gels were applied twice a day at 8 a.m. and 12 p.m., respectively, on all groups, and the diameter was measured. The gels were applied for 21 days, and the diameter was measured after 1, 3, 7, 9, 11, 19, and 21 days. The wound healing was noted for 21 days. At the 21st day, diameter was noted, and animals were sacrificed. Wound contraction rate was calculated by the formula given below [2].

% Wound contraction = A_i_ − A_t_/A_i_ × 100 (ii)

### 2.4. Histopathological Examination

After sacrificing, the burned skin area from each group was removed and dipped in 10% formalin solution. Skin was then embedded in liquid paraffin, cut into 5 μm thickness, and dried at room temperature for at least one day. Before staining, slides were kept in the oven to melt paraffin at 60 °C. The detailed process for staining slides is as follows. Slides were washed with xylene twice for 10 min each. Then, they were rehydrated via a series of ethanol solution continuously for 5 min by decreasing concentration of ethanol from 100% → 95% → 70%. Slides were washed with distilled water and stained in hematoxylin for about 8 min. Slides were then washed with tap water. In the next step, slides were immersed in 1% acid alcohol for 30 s and washed under tap water. After that, slides were dipped in ammonia solution for 30 s and washed again with tap water. Sliedes were dipped in ethanol 95% for 10 s again. Slides were then counter-stained in eosin for 1 min, and again dehydrated by serious of alcohol solution with increasing concentration from 95–100% twice for 5 min. Wash the slides with xylene twice for 5 min. Slides were then mounted on xylene-based mounting medium. The histological changes were observed under a camera-fitted microscope, and images were taken at 4X [2,25].

### 2.5. Anti-Inflammatory Studies

For inflammation, inflammatory markers, such as IL-6 and TNF-α, were determined in the blood. Blood samples were taken from each group and samples were centrifuged at 6000 rpm for 15–20 min. Serum was separated, and IL-6 and TNF-α level was determined in plasma by ELISA technique. The procedure for both IL-6 and TNF-α is same, with the exception that kits are different for both.

### 2.6. Assay Procedure (ELISA Method)

1. Add 100 μL serum from control and sample to individual well. Cover the plate with seal provided with the kit, and incubate for 90 min at 37 °C. The solutions should be added to the bottom of the micro-ELISA well plate; avoid touching the inside wall and avoid foaming as much as possible.
2. Remove liquid from each well, do not wash immediately; add 100 μL biotinylated detection Ab working solution to each well of the plate. Cover with the seal and incubate for 1 h. at 37 °C.
3. Remove the solution from each well and add 350 μL of wash buffer solution to each well. Soak for 2 min and aspirate and decant the solution from each well. Dry it completely using clean filter paper. Repeat washing three times. A micropipette washer can also be used for washing.
4. In the next step, add 100 μL HRP conjugate working solution to each well. Cover with the plate sealer and incubate for 30 min at 37 °C. Remove solution from each well and repeat the washing process five times.
5. Add 90 μL of substrate reagent to each well. Cover with new plate sealer. Incubate for 15 min at 37 °C. Protect the plate from light. Keep it for 30 min for the reaction to take place (when the actual change in color observed). However, do not keep for more than 30 min.
6. Add 50 μL of stop solution to each well.
7. Determine the optical density (OD) value of each sample by placing in micro plate reader set at 405 nm.

### 2.7. Calculation of Results

Average the duplicate readings for each standard and sample. Then, subtract the average zero standard optical density. Plot a curve with standard concentration on *X*-axis and OD values of samples on *Y*-axis. Plot the curve for standard and determine the values of IL-6 TNF-α by putting them in the equation obtained from the standard curve.

### 2.8. Statistical Analysis

Statistical analysis of data was executed by Graph pad prism 8.0. The animals were assigned in five treatment groups, each having 6 rats each. Two-way analysis of variance was applied (days × treatment), followed by *post hoc* Bonferroni test. Results are presented as mean ± SD. A value of *p* < 0.05 was accepted as significant.

## 3. Results and Discussions

### 3.1. pH

pH values of the skin ranged from 4–7 [26]. The pH values of all formulations were in an acceptable range. Mean pH values ranged between 4.65 ± 0.1014 and 5.05 ± 0.04725.

### 3.2. Conductivity

The conductivity of all gels was in the order of drug- and extract-loaded nanogels (1111 µS/cm) > drug-loaded nanogels (632 µS/cm) > extract-loaded nanogel (221 µS/cm). The increase in conductivity is associated with an increase in water contents and more polar groups/polar nature of drug [27].

### 3.3. Viscosity

The viscosity of all nanogels was in the order of drug- and extract-loaded nanogels (306.33 ± 0.577 cP) > drug-loaded nanogel [28] (222.66 ± 0.763 cP) > extract-loaded nanogel (215.33 ± 0.759 cP). The increase in the consistency of co-combination nanogels is due to the incorporation of drug and extract into the gel, which ultimately leads to an increase in viscosity [28]. An increase in viscosity is also associated with a decrease in water contents [29].

### 3.4. Spreadability

It was noticed that, as viscosity increases, spread ability decreases. This shows that spread ability is inversely proportional to viscosity. Similar results were reported by [30] in their study [30]. Spreadability of all nanogels were in the order of drug- and extract-loaded nanogels (23.93 ± 0.208%) < drug-loaded nanogels (38.6 ± 0.577%) < extract-loaded nanogels (39.52 ± 0.5%). The decrease in spreadability is due to an increase in viscosity [31]. Results are shown in Table 2.

### 3.5. Globule Size

Globule size of all active ingredients incorporated into the Carbopol gel were in the order of drug and extract-loaded gel > extract-loaded gel > drug-loaded gel. The increase in size, in the case of co-combination gel, is due to Carbopol 934 and the addition of extract in the gel matrix [32]. The globule size of all nanogels was found to be in the order of drug and extract-loaded nanogels (329 ± 0.573 nm) < extract-loaded nanogels (200 ± 0.577 nm) < drug-loaded nanogels (180 ± 0.208 nm). The increase in the size of co-combination gel is due to the incorporation of extract into the gel, along with pregabalin [33]. 

### 3.6. Zeta Potential

Zeta potential is an indication of the stability of formulation. Value ranges from 30 to 35 are considered stable. The zeta potential of all gel formulations were drug-loaded (−34.2 ± 0.1) > extract-loaded (−32.4 ± 0.1) > drug- and extract-loaded (−14.1 ± 0.1). The presence of OH^−^ ions adhering to oil/Tw80 and water film imparts negative sign to the zeta potential. These OH^−^ ions can be replaced by H^+^ ions from the medium, resulting in less negative zeta potential [34].

### 3.7. Polydispersity Index

The polydispersity index gives an indication of how uniformly the particles are distributed in a formulation. The particles are more uniform when they are closer to the zero of the PDI value [34]. The polydispersity index of nanogels was in the order of drug-loaded nanogels (0.56 ± 0.56) > drug- and extract-loaded nanogels (0.54 ± 0.01) > extract-loaded nanogels (0.34 ± 0.001). Small values indicate the narrow size distribution of the particles, as well as more uniformity and physical stability [35]. All the nanogels were found to be stable. Results are shown in Table 3.

### 3.8. Drug Contents

Drug contents were in the order of extract-loaded gel > drug- and extract-loaded gel > drug-loaded gel. Values 93 > 92 > 80 are the drug content percentage of the gels.

The zeta potential gives the indication of the stability of formulation. Values around ±30 mv draw a line between stable and unstable formulations. The zeta potentials of all gels were found to be within the range of −14.1 ± 0.1 to −34.2 ± 0.17 mv. 

### 3.9. Characterization of Gels

#### 3.9.1. Drug–Excipient Compatibility Studies

**Figure 2:**
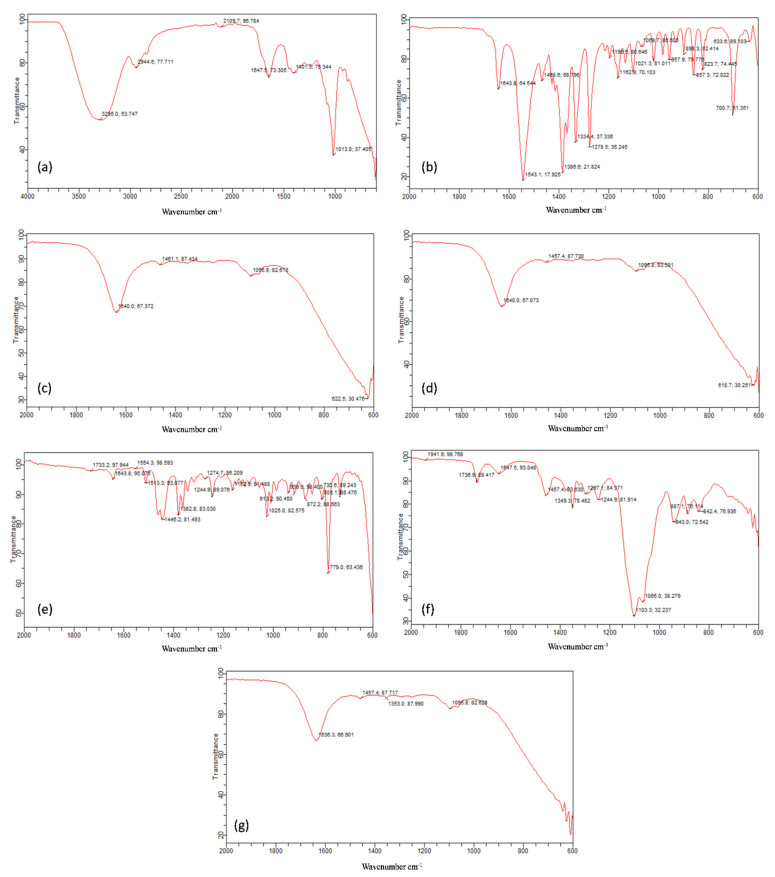
Compatibility studies between excipients and topical gels depicted through FTIR spectra of (**a**) pure extract, (**b**) pure drug, (**c**) drug and extract loaded gel, (**d**) drug loaded gel, (**e**) pure Frankincense oil, (**f**) Smix and (**g**) extract loaded gel.

The compatibility between drugs and excipients was checked by FTIR technique (Figure 2). The absorption peaks of pure drug pregabalin were scanned from 400–2000 cm^−1^. Pregabalin showed absorption bands at 1643 cm^−1^ for N-H (N-H bending), 1543 cm^−1^ for N-O (N-O asymmetric stretching), 1468 cm^−1^ for C-H (C-H bending), 1279 cm^−1^ for C-O (C-O stretching), and 860 cm^−1^ for O-H bending [36].

*Withania coagulans* extract peaks were reported in the literature at 1733, 1446, 1382, 1162, and 936 cm^−1^. The FTIR peaks of our extract sample of *Withania coagulans* appeared at 3295, 2944, 1647, 1401, and 1013 cm^−1^. The peaks between 3000–2800 cm^−1^ are due to lipids, and they are responsible for CH stretching vibrations. The band at 2944 cm^−1^ is due to the CH_2_ and CH_3_ groups; so, it can be related to alkenes group present in *Withanaia coagulans* extract. The peak at 1647 cm^−1^ is due to the stretching vibration of C=C, due to deformation of the aromatic ring of flavonoids [37]. The peak at 1013 cm^−1^ indicates the C-O structure vibrations of β-isomers of hydroxyl ketones [38].

Frankincense oil contains diterpene alcohol. The strong peak at 1162 cm^−1^ is due to the presence of lipids and alcohol (stretching of C-O bond and bending C-OH group of alcohol). Alcohol shows two bands in the 1300–1450 regions, due to bending vibration of CH_2_-CH_3_ aliphatic groups. Here, in our IR spectra of oil, this CH_2_-CH_3_ bending was observed at 1446 cm^−1^. The peak at 1733 cm^−1^ was due to carbonyl functional group of COOH [39]. Smix (Tween 80: Transcutol P) showed absorption peaks at 1736 cm^−1^ due to C=O stretching and 1647.5 cm^−1^ due to amide group [40]. The characteristics peaks of pregabalin were present in the gel formulations, as well, which indicates that there was no drug excipient interaction. The sharp peaks remained intact in the extract-loaded, pregabalin-loaded, and combination of both pregabalin- and extract-loaded nanogels.

#### 3.9.2. DSC and TG Analysis

**Figure 3:**
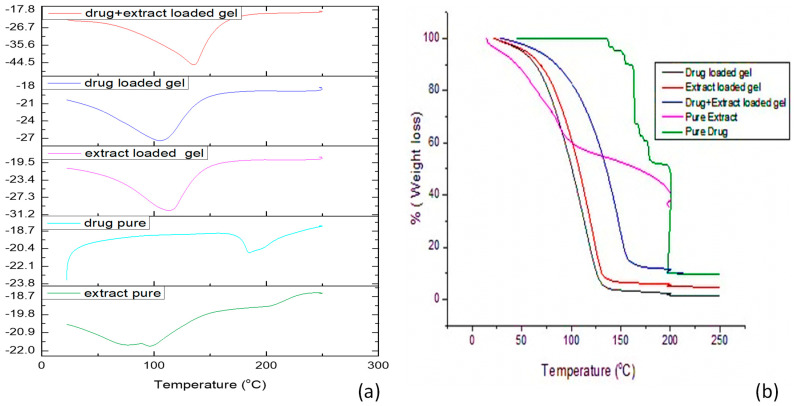
DSC (**a**) and TGA (**b**) curves of the active ingredients and prepared formulations.

The DSC curve of the pure drug exhibited a sharp endothermic peak at 196.46 °C, resultant in its melting point (Figure 3). The DSC of the *Withania coagulans* extract shows a sharp peak at 111.26 °C. Formulated gels loaded with pregabalin, *Withania coagulans* extract, and a combination of both *Withania coagulans* and pregabalin showed broader endothermic peaks. These peaks were closer to the melting point of extract. The drastic shift in the endothermic peak of pregabalin-loaded gel from 196.4 °C to 140 °C was due to the incorporation of pregabalin in the Carbopol gel. The shift/intensity of pregabalin-loaded gel was lower than that of pure pregabalin. This might be due to the incorporation of pregabalin into the gel, changing it from a more crystalline to a less crystalline/amorphous form [41]. This leads to the formation of a new peak, shifting it toward lower temperatures. Additionally, this gives an indication of the formation of a new linkage with the gel, after incorporation into Carbopol, with 934 resulting in an amorphous or disordered crystalline phase. This leads to formation of new phase, due to the low degree of crystallinity, which is due to complexation [42]. There was slight shift in the endothermic peak of *Withania coagulans*-loaded gel, which is still closer to the melting point of *Withania coagulans*.

The TGA analysis of pregabalin (Figure 3) shows no weight loss up to 205 °C [43]. At 115–111 °C, the weight loss was attributed to the desorption of surface water or decomposition of the organic contents of fruit extract of *Withania coagulans* [44]. The TGA curve for the pure drug shows that the mass remains constant with temperature, but it starts falling when it starts reaching its melting point. A similar effect was observed in all remaining gels. This study confirms that the excipients and moisture contents have no adverse effects on formulations [41]. There was a slight change in the peak, from 115.26 to 126 °C. In the case of the extract-loaded gel, the weight loss was found to be 1.05%, which may be associated with the decomposition of the organic contents of the extract. Similarly, weight loss from the TGA curve, in the case of the drug-loaded gel and combination of extract and pregabalin-loaded gel, was found to be 1.37% and 0.49%, respectively.

#### 3.9.3. X-ray Diffraction of Gels

**Figure 4:**
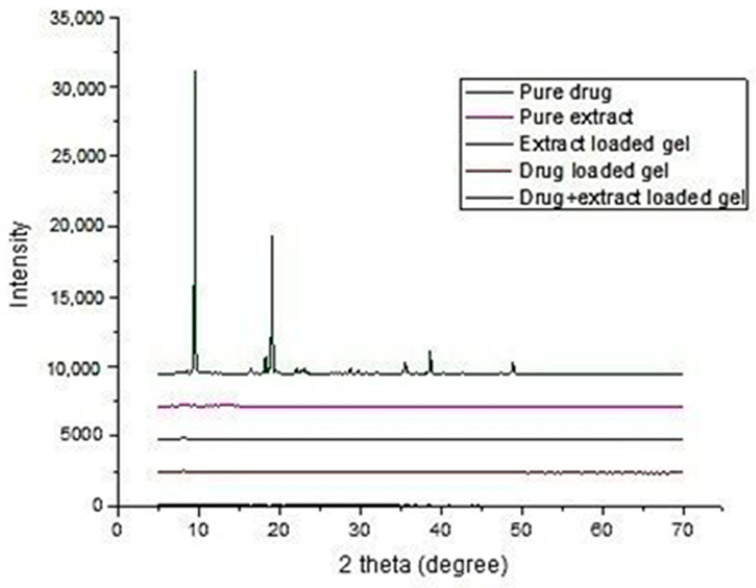
Graph between intensity and 2 θ° of all formulations.

The X-ray diffractogram of pregabalin confirms its crystalline nature, as evidenced by the number of sharp and intense peaks (Figure 4). The XRD pattern of pure pregabalin at 2θ shows characteristic peaks at 9.4, 19.04, 38.5, 40, and 49.9 [45].

The X-ray diffractrometer of *Withania coagulans* and all three gels pregabalin-loaded, *Withania coagulans*-loaded, and drug- and extract-loaded gels confirms the amorphous nature, due to the absence of sharp peaks [46]. It was clear that pregabalin characteristic peaks were modified in the same thermal events, suggesting a partial amorphous nature in all the gels. These results were consistent with the results reported by [47].

#### 3.9.4. Transmission Electron Microscopy (TEM)

**Figure 5:**
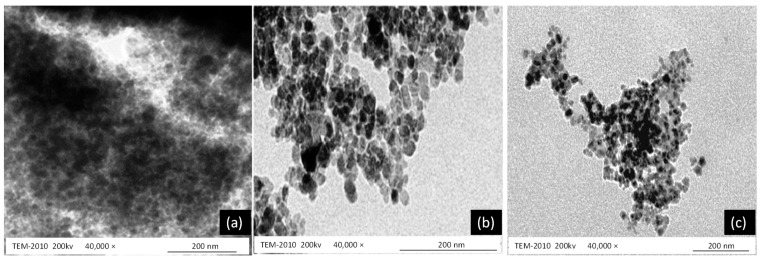
Shows TEM images of extract-loaded gel (**a**), pregabalin-loaded gel (**b**), and co-combination of pregabalin and extract (**c**).

TEM images (Figure 5) of the extract-loaded gel (a), pregabalin-loaded gel (b), and co-combination of pregabalin and extract (c). TEM image (a) of *Withania coagulans* extract-loaded gel showed that particles were spherical in shape and atypical, with a size of 200 nm, whereas the pregabalin-loaded gel shows that particles were spherical in shape, with a size 200 nm. Co-combination of pregabalin with *Withania cogulans* also depicted the spherical shape of particles, with a particle size of 200 nm. This shows that the particles slightly decreased, due to the incorporation of the extract and drug in Carbopol, as compared to the drug alone. This shows that the interaction and linkage take place between the drug and pregabalin, resulting in a decrease in size, which indicates that conjugation has taken place between the drug and extract, resulting in the formation of co-combination gel [48]. The size of *Withania coagulans* particles alone is larger (200 nm) than pregabalin; however, when it is incorporated into the Carbopol gel, along with pregabalin, its size decreased, showing complete bonding with the drug and gel (200 nm). This decrease in size might be associated with a reduction in the mobility of surfactant [32]. Similar results were proposed by [37]. These TEM results are co-related with the results obtained by the zetasizer.

### 3.10. Wound Contraction Rate

The wound area was captured, and the wound healing rate was assessed. It was observed that wound healing percentage increases in a time-dependent manner, as mentioned in the graph below.

**Figure 6:**
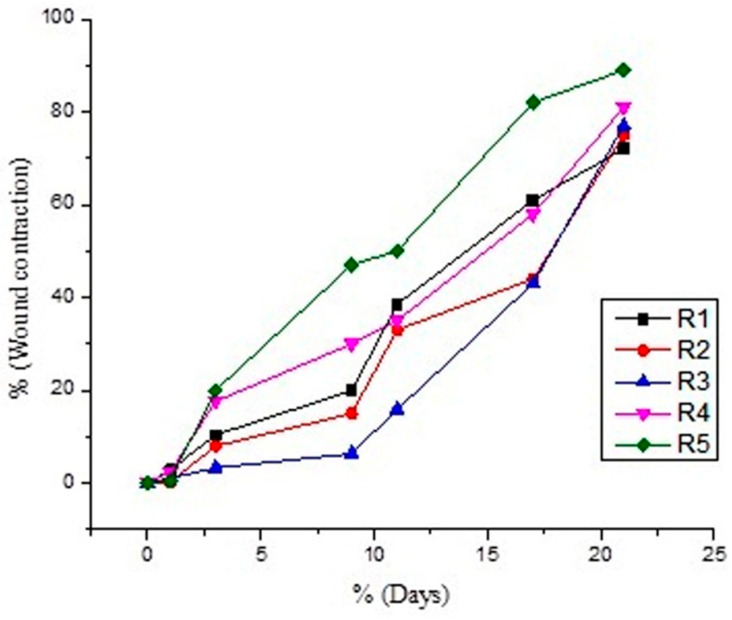
Graph between wound contraction rate and time.

**Figure 7:**
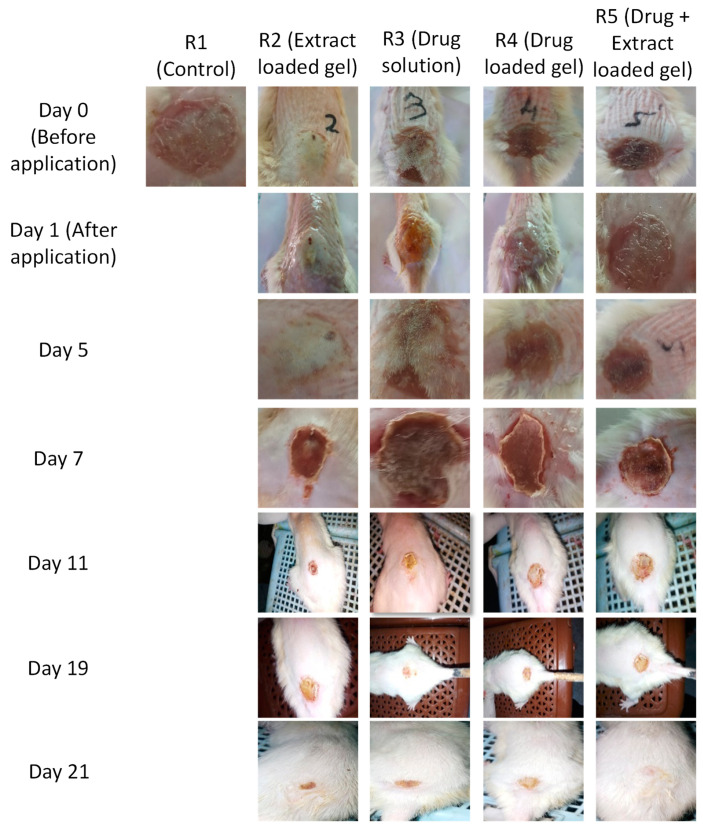
Effect of different nanogels on wound contraction in rat models.

Percent decrease was in the order of R5 (89%) > R4 (84%) > R3 (81%) > R2 (78%) > R1 (71%). Decrease in diameter was noted after the application of gels to each group for a period of 21 days. Results were compared with the control. It was noted that the combination of pregabalin and *Withania coagulans* extract-loaded gels show a maximum decrease in diameter after 21 days. The order of healing in burn injury in different animals group was: combination (pregabalin and *Withania coagulans* extract-loaded gel) > *Withanis coagulans-*loaded gel > drug-loaded gel > drug solution > control (no treatment). This shows that the combination of pregabalin and *Withania coagulans*-loaded gels is effective in topical burn injury treatment. Results are shown in Table 4 and Figure 6 and Figure 7.

Percent wound contraction in burn wound injury rat models, using different formulations, was no observed throughout the period of study (21 days). It was observed that there was a significant reduction in the wound area from day 1 to day 21. In comparison to the control group, where no treatment was applied on the wound, the treated groups show significant reduction in wound area and healing. The order of % wound area contraction was in following order: R5 (89.9%) > R4 (84.7%) > R3 (81.3%) > R2 (78.8%) >R1 (71.1%). ANNOVA gives significant results in all formulation, with a *p* value < 0.0001.

Multiple comparison models of all formulations show a significant reduction in wound area, with the passage of days from day 1 to day 21. All formulation give *p* value < 0.0001, in comparison to control group R1. The trend remains to be significant in all cases, and the wound contraction rate was at maximum in the case of co-combination of pregabalin and *Withania coagulans* extract-loaded gel.

### 3.11. Histopathological Examination

**Figure 8:**
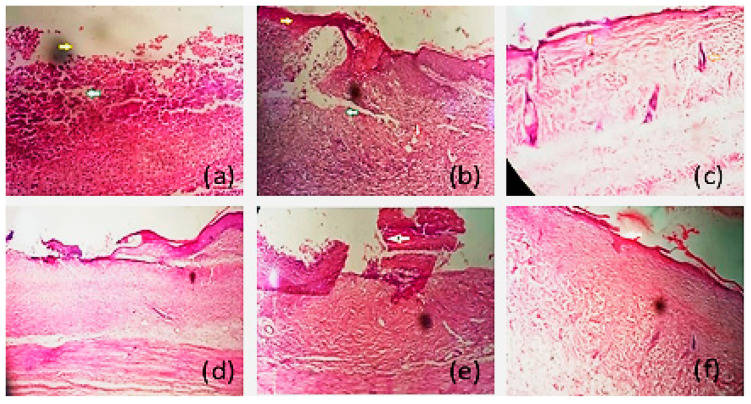
Histopathological examination of different formulations, skin denudation, and inflammation of R1 (**a**,**b**), skin regeneration of R2 (**c**), fibrosis of R3 (**d**), fibrosis and regeneration of R4 (**e**), and skin epithelization of R5 (**f**).

In cases of third degree burn or deep thickness injury, the thermal injury extends to hair follicles and destroys them, thus decreasing their capacity to regenerate epidermis. Additionally, still-present necrosis may become the cause of infection, as healing time taken is prolonged [49]. Extensive activity of macrophages is required, in order to remove them [50]. Histopathological examination of skin of all animals group is shown in Figure 8 below.

Animals in R1 were the control group receiving no treatment. Histopathological examination of R1 group shows that there is complete removal of epidermis, and skin denudation is shown by yellow arrows in Figure 8a,b. The green arrow in the figure shows the inflammatory cells, and the red arrow shows that the macrophages started accumulating. In our study, histopathological examination shows that, in the case of group R1, there was complete damage of epidermis, including all its layers. Dermis was also damaged to some extent, but the basal layer was intact. There was a presence of inflammatory marker-like mast cells in the dermis; vasculization and fibrosis were also present. There was presence of adipose cells in the dermis, as well, for healing at the local site

After 21 days of treatment with different formulations, histopathological examination shows the healing processes of the different groups, as indicated in Figure 8 below.

The R2 group (Figure 8c) includes the animals treated with *Withania coagulans* extract-loaded gel. Histopathological examination showed that skin starts regenerating, as there was epithelization of epidermis and hairs follicles present, as indicated by the orange arrow. In the case of the R2 group, there was the formation of a keratin layer at the top of the epidermis. The components of dermis were intact, and there was the presence of hair follicles and sweat glands in the dermis, as well. Adipose cells were also found.

The R3 group of rats was treated with pregabalin solution (Figure 8d). It was noted that fibroblast cells were present, but there was still skin denudation. Skin generation was not proper, as there was neither epidermis nor dermis regeneration. Fibrosis can be seen in the epidermis and dermis. Skin was not regenerated.

R4 was the group of rats treated with pregabalin gel. It was clearly indicated in the Figure 8e that fibrosis existed, and the skin healing process was started. In the case of the fourth group, R4, it was noticed that there was no keratin layer on the top of epidermis. The process of fibrosis took place, and the dermis was not well-regenerated. There were no hair, follicles, or sweat glands seen in the dermis layer

The R5 group was treated pregabalin and *Withania coagulans* extract-loaded gel (Figure 8f). There was complete or proper skin reepithelization. A thin layer of skin can be seen, as indicated by white arrow. Dermis was filled fibroblast and blood vessels, indicating a well-developed granulation tissue formation. In the case of group R5, proper skin regeneration, found as proper stratified epidermis, can be seen. Dermis cells starts regaining their original position after injury following the application of formulation, showing that the healing process was going on smoothly. Markel cells presence indicate the healing process.

These histopathological studies showed that co-combination gel succeeded in regenerating the top layer of the skin. The regeneration of keratin layer was thicker in the case of the R5 group, showing good regeneration of skin. Wound healing was also found to be satisfactory in the case of R2, where skin regeneration can be seen, but it was a thin layer. This study showed that pregabalin and *Withania coagulans* combination can be used for healing burn-induced injury. The results of *Withania coagulans* gel and pregabalin alone were also satisfactory. Topical application pregabalin drug solution alone did not show great results, in comparison to the combination gel. As wound contraction was also found to be at maximum, in the case of co-combination gel, then either the gel alone or control group.

### 3.12. Statistical Analysis

Percent wound contraction in Burn wound injury rat models using different formulations was no observed throughout the period of study (21 days). It was observed that there was significant reduction in wound area from day 1 to day 21. In comparison to the control group, where no treatment was applied on the wound, the treated groups showed a significant reduction in wound area and healing. The % of wound area contraction was in following order: R5 (89.9%) > R4 (84.7%) > R3 (81.3%) > R2 (78.8%) >R1 (71.1%). ANNOVA gives significant results in all formulation, with *p* value < 0.0001.

Multiple comparison models of all formulations showed a significant reduction in the wound area, with the passage of days from day 1 to day 21. All formulation gave *p* value < 0.0001, in comparison to the control group (R1). The trend remains to be significant in all cases, and the wound contraction rate was at maximum in the case of co-combination of pregabalin and *Withania coagulans* extract-loaded gel.

### 3.13. Anti-Inflammatory Activity IL-6 and TNFα

**Figure 9:**
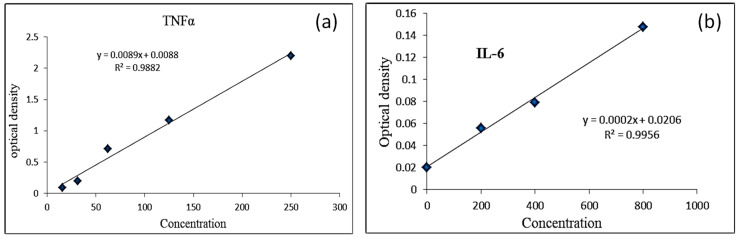
Standard curve of (**a**) TNFα and (**b**) IL-6.

Calibration curve for IL-6 and TNF-α Standard. Results were calculated from the equation obtained from the standard curve for both TNFα and IL6 as shown in Table 5, Table 6 and Figure 9 below. After putting the values in the equation, it was found that TNF-α value was lower, in the case of co-combination gel (132.2 pg/mL), as compared to the control (140.22 pg/mL). Similarly, in the case of IL-6, the value was found to be (78 pg/mL) in the case of co-combination gel and 81 pg/mL in the case of the control. This result shows that co-combination gel is effective in reducing inflammation and burn-induced injury.

## 4. Conclusions

The aim of current study was to check the effectiveness of plant extract (*Withania coagulans*)-loaded, along with a drug (pregabalin), topical gels on burn injury. We successfully prepared the nanogel and analyzed its effectiveness on a rat model via infliction of a burn wound. The outcomes indicated that the combined gel using pregabalin and plant extract reduced the wound surface area effectively, with a wound contraction rate of 89.95%. Anti-inflammatory studies further indicated that the IL-6 and TNF-α value were 132.2 pg/mL, as compared to the control (140.22 pg/mL). Similarly, the IL-6 value was found to be 78 pg/mL for co-combination gel and 81 pg/mL in the case of the control. Histopathologically, co-combination gel also heals wounds more quickly, compared to individual gel, deeming it effective for biological systems. Plant extract can be used successfully along with other drug for different skin diseases in the future, as a safer remedy. Human studies using co-combination gel can be carried out for effective results. Assessment for other formulation components (oil, surfactants/co-surfactants, gelling agents, and other plant extract) can be used. Other methods for burn treatment can be used, following co-combination therapy. Studies can be extended to the molecular level for a detailed insight.

## Figures and Tables

**Table 1:** Composition of pregabalin and *Withania coagulans* topical gel includes frankincense oil, Transcutol P: Tween 80 (1:1), and water.

Formulation	Active %	Smix (Transcutol P: Tween 80 (1:1))	Frankincense Oil	Water	Carbopol 934
Pregabalin	2.5%	50%	15%	35%	1%
*Withania coagulans*	2%	50%	15%	35%	1%
Pregabalin + *Withania coagulans*	2.5% + 2%	50%	15%	35%	1%

**Table 2:** Physico-chemical properties of all three topical gels.

Sr.no	pH ± SD	Conductivity ± SD mS/cm	Viscosity ± SD cP	Spreadability ± SD%	Drug Contents (%) ± SD
Drug-loaded gels	4.65 ± 0.1014	632 ± 1	222.66 ± 0.763	38.6 ± 0.577	92 ± 0.644
Extract-loaded gels	5.05 ± 0.04725	221 ± 1	215.33 ± 0.759	39.52 ± 0.5	93 ± 0.680
Drug- and extract-loaded gels	4.836 ± 0.0321	1111 ± 1	306.33 ± 0.577	23.93 ± 0.208	80 ± 0.661

**Table 3:** Globule size, polydispersity index, and zeta potential of topical gel.

Sr.no	Globule Size ± SD nm	PDI ± SD	Zeta Potential ± SD mv
Drug-loaded gels	180 ± 0.208	0.56 ± 0.56	−32.4 ± 0.1
Extract-loaded gels	200 ± 0.577	0.34 ± 0.001	−34.2 ± 0.17
Drug- and extract-loaded gels	250 ± 0.573	0.54 ± 0.01	−14.1 ± 0.1

**Table 4:** Wound surface area (mm) changes at measurement days of different groups, n = 6.

Days	R1 (Control)	R2 (*Withania coagulans* gel)	R3 (Pregabalin Solution)	R4 (Pregablin gel)	R5 (Pregabalin + *Withania coagulans* gel)
0	18.99	17.79	19.03	16.82	20.98
1	18.45	17.37	18.98	16.62	20.85
3	17.01	14.65	17.35	16.25	16.75
9	15.21	12.52	15.99	15.82	11.06
11	11.67	11.43	12.60	14.14	10.35
19	7.23	7.33	10.5	9.51	3.69
21	5.20	3.36	4.76	3.82	2.28

**Table 5:** IL-6 and TNF-α standard optical density against different concentrations.

IL-6	Average Abs	SD	TNF-α	Average Abs	SD
1.202	1.18633333	0.01464013	0.181	0.17766667	0.0057735
1.184	-	-	0.181	-	-
1.173	-	-	0.171	-	-
Control	Average	SD	Control	Average	SD
1.258	1.25533333	0.00305505	0.186	0.183	0.00519615
1.256	-	-	0.186	-	-
1.252	-	-	0.177	--	

**Table 6:** IL-6 and TNF-α result of co-combination gel (E+D).

Sr. no	IL-6		TNF-α	
	Conc.	OD	Conc.	OD
1	00	0.02	250	2.202
2	200	0.056	125	1.169
3	400	0.076	62.5	0.847
4	800	0.128	31.25	0.173
5	1000	0.243	15.625	0.095

## Data Availability

Most of the data has been presented in the main article. Raw data to reproduce these findings cannot be shared at this time due to technical and time limitations.
